# Functional and Bioactive Properties of Peptides Derived from Marine Side Streams

**DOI:** 10.3390/md19020071

**Published:** 2021-01-29

**Authors:** Ilknur Ucak, Maliha Afreen, Domenico Montesano, Celia Carrillo, Igor Tomasevic, Jesus Simal-Gandara, Francisco J. Barba

**Affiliations:** 1 Faculty of Agricultural Sciences and Technologies, Nigde Omer Halisdemir University, 51000 Nigde, Turkey; malihaafreen120@gmail.com; 2 Department of Pharmaceutical Sciences, Section of Food Sciences and Nutrition, University of Perugia, Via S. Costanzo 1, 06126 Perugia, Italy; domenico_montesano@yahoo.it; 3 Nutrition and Food Science, Faculty of Science, Universidad de Burgos, 09001 Burgos, Spain; ccarrillo@ubu.es; 4 Department of Animal Source Food Technology, Faculty of Agriculture, University of Belgrade, Nemanjina 6, 11080 Belgrade, Serbia; tbigor@agrif.bg.ac; 5 Nutrition and Bromatology Group, Department of Analytical and Food Chemistry, Faculty of Food Science and Technology, University of Vigo, Ourense Campus, E32004 Ourense, Spain; jsimal@uvigo.es; 6 Department of Preventive Medicine and Public Health, Food Science, Toxicology and Forensic Medicine, Faculty of Pharmacy, Universitat de València, Avda. Vicent Andrés Estellés, 46100 Burjassot, Spain

**Keywords:** bioactive peptides, seafood side streams, functional properties, antioxidant, antihypertensive, nutraceuticals

## Abstract

In fish processing, a great amount of side streams, including skin, bones, heads and viscera, is wasted or downgraded as feed on a daily basis. These side streams are rich sources of bioactive nitrogenous compounds and protein, which can be converted into peptides through enzymatic hydrolysis as well as bacterial fermentation. Peptides are short or long chains of amino acids differing in structure and molecular weight. They can be considered as biologically active as they can contribute to physiological functions in organisms with applications in the food and pharmaceutical industries. In the food industry, such bioactive peptides can be used as preservatives or antioxidants to prevent food spoilage. Furthermore, peptides contain several functional qualities that can be exploited as tools in modifying food ingredient solubility, water-holding and fat-binding capacity and gel formation. In the pharmaceutical industry, peptides can be used as antioxidants, but also as antihypertensive, anticoagulant and immunomodulatory compounds, amongst other functions. On the basis of their properties, peptides can thus be used in the development of functional foods and nutraceuticals. This review focuses on the bioactive peptides derived from seafood side streams and discusses their technological properties, biological activities and applications.

## 1. Introduction

The aquatic ecosystem with its great biodiversity is a reservoir of millions of species containing nutrients and little utilized biologically active compounds (BACs) of potential high added value for food, pharmaceutical and cosmetic applications, among others. Extensive research efforts are being conducted to dive into and learn more about the unexplored opportunities for biotechnology in the seas.

The worldwide fish production in 2016 was around 171 million tons, with 91 million tons coming from inland and marine fisheries, and approx. 80 million tons from aquaculture [1]. Fish processing generates a great amount of side stream biomass composed of heads, skins, viscera, backbone, trimmings and blood, which is estimated to range from 25 to 70%. According to data from the FAO, approx. 28 million tons of accessible refuse is generated on a global scale [1]. The disposal of these products remains a difficult problem because if abandoned in the environment, they can generate serious alerts, and their use in aquaculture or as feed is not always viable or advantageous. However, it must be considered that fish side streams contain multiple ingredients of high biological and nutritional value, such as fish oils, proteins and peptides, collagen, gelatin, enzymes, chitin and minerals. From this point of view, therefore, they could constitute a low-cost opportunity for the development of functional foods and nutraceuticals, as well as pharmaceutical and cosmetic health products. At the same time, a massive and rational use of waste and low-value seafood could effectively contribute to improving the environmental and ecological sustainability of production [2].

Among the different ingredients that can be recovered from fish side streams, fish peptides have interesting functional properties for food applications, such as being easily soluble and having emulsifying, foaming and gelling properties, which contribute to product consistency and shape [3]. Moreover, apart from the functional properties attributed to fish peptides, many medicinal and therapeutic properties have also been reported for these bioactive compounds [4,5]. Among the various relevant activities ascribed to these peptides, noteworthy are the antioxidant, antihypertensive, antimicrobial, antithrombotic, immunomodulatory and opioid-like activities [6]. Some studies have also found that bioactive peptides can be used in the prevention of cancer as they have the ability to stop cancer-causing agents [6].

These peptides are short sequences of two to 20 amino acids, which can be obtained from a wide variety of fish side streams since protein is one of the major constituents in fish. Among the different parts of fish, muscles contain about 17–22% protein, while other body parts contain 8–35% protein [7,8]. Muscle proteins are abundantly present in different fractions of fish side streams and are highly nutritious, providing both essential and non-essential amino acids. In fact, their nutritional value is similar to that of meat and egg proteins and, in particular, the high contribution, compared to meat, of the essential amino acids lysine, methionine and threonine is of great interest. Furthermore, these proteins have several beneficial metabolic effects such as the regulation of glucose metabolism, a positive influence on the lipid profile and the regulation of blood pressure [9,10]. Muscle proteins, in the form of protein hydrolysates, are used in the food industry and therapeutics as a good source of bioactive peptides with technological and physiological properties [11]. Next to muscle protein, peptides obtained from collagen recovered from skin and bone side streams have also been reported for their health-related properties [12,13,14]. Moreover, fish intestine, gills, heads, viscera, frames or trimmings have been also reported as good sources of bioactive peptides [15,16,17,18].

Fish side stream biomasses are currently used to produce protein hydrolysates, containing essential and non-essential amino acids, bioactive peptides and proteoglycans [7]. There is increasing interest in several bioactive peptides with documented bioactivity [4] and, as a result, the use of these substances as nutraceuticals and functional foods in health is increasingly growing and widespread [19]. For instance, at this moment, on the market there are peptides obtained from the enzymatic hydrolysis of proteins assisted by the enzymes alkalase, trypsin and chymotrypsin [20], although there is still a limited exploitation of the application of peptides in food [6,21,22]

**Figure 1:**
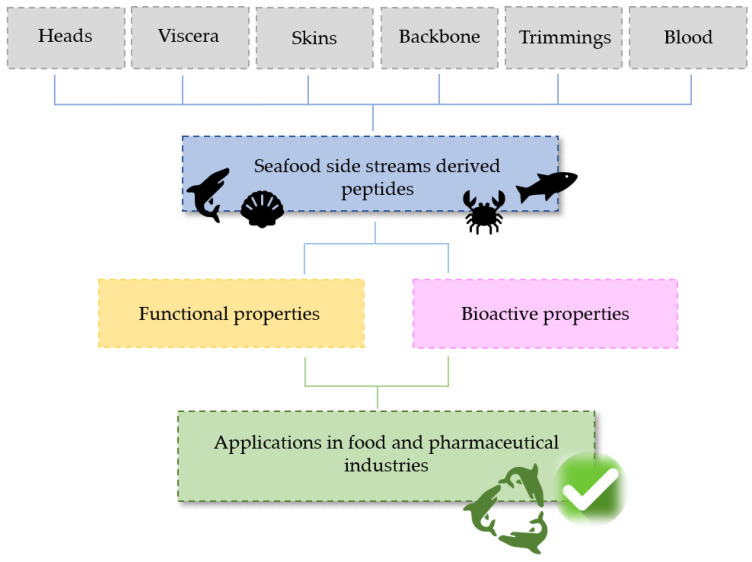
Sources of seafood bioactive peptides, properties and applications.

Therefore, taking into account the great potential for the commercial exploitation of fish side stream biomass-based proteins and their hydrolyzed forms, the objective of this study is to review the available methods for the recovery of peptides from fish by-products, their functional properties and bioactivity and the existing sources, as well as the potential applications of these bioactive compounds. In Figure 1, an overview of the main sources of seafood-derived bioactive peptides, their properties and major applications is presented.

## 2. Methods to Separate Peptides from Seafood Side Streams

Although naturally active peptides can be found in the food matrix, as long as they are embedded in the proteins, these bioactive compounds are usually obtained after protein breakdown. It should be noted that in the form of protein, these peptides do not have any function, so protein breakdown is necessary to change its sequence into the form of active peptides.

Different methods have been established to break down protein, including chemical and enzymatic hydrolysis or microbial fermentation (for a review, see [23]). Moreover, gastrointestinal digestion or food processing can also release these bioactive compounds [23]. The extraction method and conditions applied, next to the amino acid composition and peptide structure, play a key role in the final properties and subsequent functionality of the peptide recovered [23].

The acid extraction technique has been used to obtain antimicrobial peptides from red sea bream gill filaments [15]. Moreover, acidification treatment has also been used to extract collagen peptides as well as to dissolve them without changing their triple helix structure. On the other hand, collagen is converted into gelatin by heat treatment which breaks covalent and hydrogen bonds and changes the helical form of collagen into the coiled form of gelatin [23]. Although chemical methods are low cost and relatively easy to operate, the lack of control over the procedure, which results in the heterogenous yield of peptides, makes enzymatic hydrolysis the most appropriate and common technique to produce bioactive peptides. The breakdown of fish body parts rich in proteins can be carried out by using variable sources of proteolytic enzymes, coming from animals, plants or microbes [24], which give end product peptides with high biological value. In this regard, the enzymes pronase, bromelain, protease A and N, orientase, neutrase, protamex, validase, pancreatin and flavourzyme are the most commonly used [4]. If enzymes promote protein breakdown in a precise way, short chains of polypeptides are produced. These polypeptides can change and expand their efficiency in various processes. Moreover, sequential enzymatic digestion is commonly used to get functionally active peptides. According to previous studies, antioxidant peptides are formed by the breakdown of many fish proteins by using industrially available proteases, like flavourzyme and alkalase [4,25,26]. For instance, enzymatic hydrolysis was used to produce antioxidant peptides from tuna backbones. In the work performed by Je et al. [27], various enzymes, such as alkalase, a-chymotrypsin, neutrase, papain, pepsin and trypsin, were used separately, with their optimal pH and temperature conditions, and the resulting peptides showed significant antioxidant action using a lipid peroxidation inhibition assay in a linoleic acid emulsion model system.

As mentioned before, bacterial enzymes can also release peptides through their own natural metabolism. With respect to this, protein hydrolysates with reported functional and bioactive properties have been prepared from Atlantic salmon side streams by a lactic acid bacteria inoculum [28].

Although fish protein hydrolysates could themselves exert biological activities, their bioactive properties are increased after a purification procedure. Therefore, after the extraction of the protein hydrolysate, fractionation and purification steps are highly recommended.

The isolation of the extracted protein hydrolysates can be performed based on their molecular weights by means of ultrafiltration membrane systems [23]. For instance, bioactive peptides have been recovered from tuna dark muscle after enzymatic hydrolysis in a system that uses a multistep recycling membrane reactor combined with an ultrafiltration membrane system [27,29].

**Figure 2:**
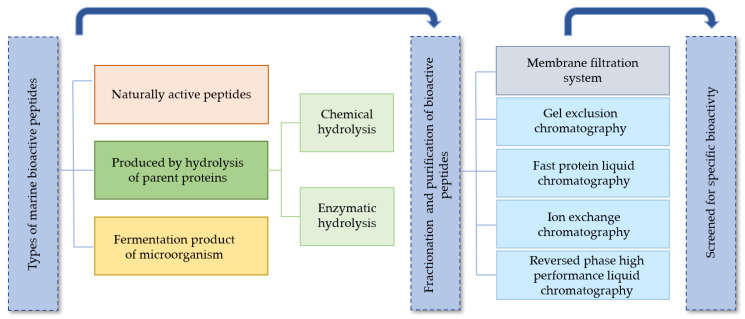
Schematic representation of the extraction and purification of marine bioactive peptides.

Gel exclusion chromatography, fast protein liquid chromatography, ion exchange chromatography and reversed phase high-performance liquid chromatography are usually the techniques applied for the purification step performed before the screening for bioactivity prior to the application of peptides in food or therapeutic products. In Figure 2, a schematic representation of the main strategies followed for the extraction, fractionation and purification of marine bioactive peptides is shown.

## 3. Functional Properties of Seafood Side Stream-Derived Peptides

**Figure 3:**
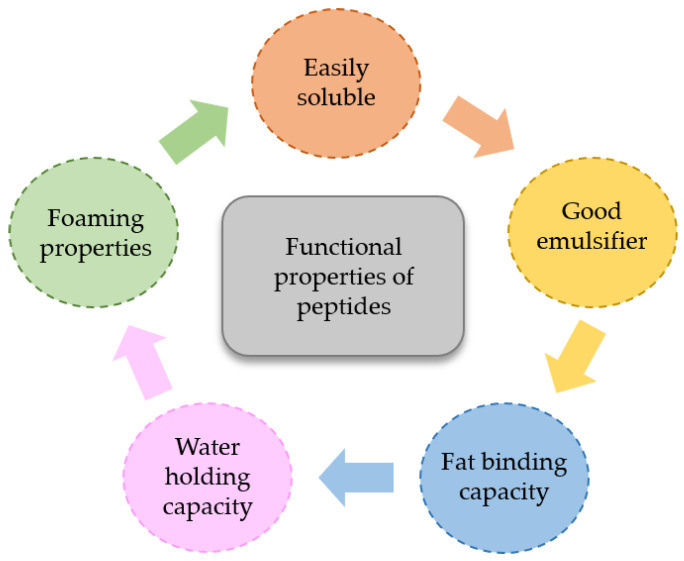
Functional properties of seafood-derived peptides.

A detailed description of the functional properties of marine-derived peptides is given below and schematically represented in Figure 3.

### 3.1. Solubility

Solubility stands out among the functional properties of protein hydrolysates. Other properties associated with these products, such as emulsification, foaming and water-holding capacity, are affected by solubility. Fish protein hydrolysates are soluble over a wide range of pH [30,31,32,33], which makes them useful for many applications in the food industry. The solubility of peptides can be affected by different things, such as the net charge of peptides. Protein hydrolysates show the lowest solubility at the isoelectric point; as pH moves away from the isoelectric point, the net charge of peptides increases, and thus higher solubilities are reached [30]. In this regard, studies on sardinella by-products [31] and tilapia fish protein hydrolysates [32] showed excellent solubilities as long as pH increased. In addition, the balance of hydrophilic and hydrophobic forces of peptides is a key determinant of peptide solubility [30]. Furthermore, the degree of hydrolysis (DH) represents a relevant parameter able to affect the solubility of protein hydrolysates. Generally, the breakdown of proteins to smaller peptides results in more soluble products. One study compared the solubility of peptides from ornate threadfin bream with different DH (10–30%) and found that the effect of the DH depended on the pH. At pH 5 and 9, the solubility increased with increasing DH from 10% to 20%, whereas no differences among the different hydrolysates were observed at pH = 7. A lower solubility was observed at pH = 5 and DH = 10%, suggesting that high molecular weight peptides precipitated at this pH, which was near the isoelectric point [33].

### 3.2. Emulsifying and Foaming Properties

Peptides have been reported to increase foam and emulsion formation in comparison with native proteins. The mechanism behind their emulsifying properties is related to the adsorption of peptides on the surface of oil droplets formed during homogenization next to the generation of a protective membrane responsible for the inhibition of the oil droplet coalescence [34]. With regard to the emulsifying properties of fish protein hydrolysates, a clear dependence on the surface properties or on how the bioactive compound decreases the interfacial tension between hydrolytic and hydrophobic constituents in food products has been reported [35].

Many studies showed that peptides had from 23 to 240% foaming ability, but those with only 20 to 140% can also have foaming properties [36,37]. The pH can also affect the foaming factor. For instance, at pH = 4, foaming activity is low but it can be maintained at a pH from 6 to 10 [30]. Molecular weight also changes the foaming activity of peptides. It was documented that molecules cannot properly align if peptides have low molecular weight, approximately 1 kDa. On the other hand, many other studies reported that a short bioactive peptide length can reduce foaming quality [38].

### 3.3. Water-Holding and Fat-Binding Capacity

As mentioned before, the water-holding capacity of fish protein can be affected by solubility. It was proved that water-holding ability decreases with high solubility [35]. Fish peptides have shown excellent water-holding capacity, ranging from 2.47 to 6.60 mL/g [30]. The water-holding capacity of peptides can be affected by different factors. In this regard, a higher number of polar groups (i.e., COOH and NH_2_), which are usually produced after enzymatic hydrolysis, has a significant effect on the amount of water absorbed. In this regard, one study on rainbow trout peptides showed that they have considerable water-holding ability because of existing amino acids (glutamic and aspartic acid) with polar side chains [30].

The physical envelopment of oil causes fat absorption, so absorption is increased by greater compactness of the protein. The fat absorption ability of peptides varies from 1.0 to 10.8 mL/g [38,39]. One work on peptides of blue wing sea robin showed that the fat-binding ability of peptides was reduced with higher molecular weights of peptides [35]. These fat-binding peptides are very important in meat and sweet industries because their ability to absorb fat can provide good taste to food [30].

## 4. Bioactive Properties of Seafood Side Stream-Derived Peptides

Biologically active compounds, also known as “bioactive compounds”, show beneficial effects on health [40]. Among the different BACs, we can find biological active peptides, which are not active when they are enclosed in proteins, but they exert their bioactivity through protein breakdown, as previously detailed [4].

There are several studies in the available literature describing the different bioactive peptides obtained from sea rotifers, cod backbones, tilapia protein, congerel, sardina, tuna and shark muscle, squid by-products, shrimp shell waste, skate (*Okamejei kenojei*) gelatin, tuna heads and bones, Atlantic salmon skin, bone and muscles, skin gelatin of seabass, unicorn leatherjacket, Alaska pollock skin collagen and salmon skin collagen, among other marine side streams [20,21,22,27,41,42,43,44,45,46,47,48,49,50,51,52,53,54,55,56]. The profile and amount of bioactive peptides and their subsequent biological activity depend on the matrix. Some examples of the health-promoting properties of bioactive peptides obtained from marine side streams are presented in Table 1.

### 4.1. Antioxidant Activity

Antioxidant peptides play a key role in preventing the oxidation of biological components [57]. Many seafood-derived antioxidant peptides have been reported (Table 1). For instance, antioxidant peptides obtained from the gastrointestinal digestion of hoki frame protein were isolated [17]; the authors observed that peptides derived from gelatin had an exclusive arrangement of amino acids with a repetitive sequence of Gly-Pro-Ala which was associated specifically with antioxidant properties. In another study, a dipeptide (Met-Tyr) derived from sardine muscle protein was isolated and it was shown to be a stimulator for the heme oxygenase-1 (HO-1) antioxidant defence protein in a concentration-dependent manner [58]. Earlier experiments observed that ferritin is necessary to promote the activity of HO-1 protein and demonstrated that the isolated dipeptide acts on both HO-1 and ferritin protein in endothelial human cells [59]. Moreover, research focused on studying the relationship between the structure and the antioxidant properties of bioactive peptides demonstrated that elements, sequence and hydrophobic amino acids influenced the function of peptides as antioxidants [60]. Peptides with antioxidant activity mostly contain amino acids with a hydrophobic nature and sequences that are shorter in length, about 5–16 amino acids, with valine and leucine at the N-terminus and proline, tyrosine or histidine also present in their sequences. Tyrosine works as a strong electron contributor because of the phenolic side chains found in its structure, so it works as significant free radical scavenger and stops the continuous reaction of radicals [61]. The molecular weight of these antioxidant peptides coming from fish protein varies from 0.5 to 1.5 kDa [33,62]. For instance, it was shown that natural peptides with a shorter sequence and smaller molecular weight worked by donating electrons, thus reacting with unrestricted flowing radicals and stopping chain elongation [36]. Those peptides whose amino acids have a more hydrophobic nature and containing sulfur had more solubility and worked as strong antioxidants [62].

### 4.2. Antihypertensive Activity

Angiotensin-converting enzyme (ACE) catalyzes specific reactions involved in blood pressure regulation, which makes this enzyme responsible for hypertension and a key target in the therapy of such a risk factor of cardiovascular disease. Antihypertensive peptides obtained from seafood have been extensively reviewed elsewhere [63], demonstrating that the recovery of these bioactive compounds is an efficient way of using seafood by-products. For instance, some studies have shown that peptides obtained through collagen hydrolysis from skate skin and sea cucumber can inhibit the activity of angiotensin-converting enzyme (ACE) [12,13,14], being a natural and potentially healthier alternative to synthetic ACE inhibitors available on the market. Moreover, squid and salmon by-products, tuna frame and Pacific cod skin have also been reported as sources of antihypertensive peptides (Table 1). Although the repetitive amino acid sequence glycine–proline–alanine present in peptides of gelatin were correlated with antihypertensive activity, peptides with different amino acid compositions have been reported for their antihypertensive activity, depending on the part of the fish, fish species or procedure applied for the peptide recovery [64]. However, there is a trend towards a higher ACE antihypertensive effect with lower molecular weight peptides. Interestingly, an efficient control of angiotensin-I-converting enzyme can be expected if the activity of peptides is monitored through quantitative structure–activity relationship modeling. In this regard, ACE inhibition of peptides has been reported to correlate with structural properties such as hydrophobicity and positive charge [65]. It was also observed that peptide sequence on the C-terminus can affect angiotensin-converting enzyme function. For example, it was recommended that lysine, arginine or proline are ideal amino acids present at the C-terminus and participate in increasing the power of ACE-I inhibition [66]. Researchers also studied peptides in spontaneously hypertensive rats and they found that if dipeptides had a tyrosine at the C-terminus, they can reduce systolic blood pressure for a long time but in a sluggish way. On the other hand, if tyrosine is replaced by phenylalanine at the C-terminus, the systolic blood pressure will decrease rapidly but only for a short time [67]. Interestingly, next to the role of marine peptides in hypertension, recent studies have also reported that they could represent a promising approach in the intervention and prevention of Severe Acute Respiratory Syndrome (SARS) virus infection, through their ACE-II inhibitory properties [63].

### 4.3. Antimicrobial Activity

Many antimicrobial bioactive peptides are obtained from seafood. In fact, the main classes of antimicrobial peptides, such as defensins, cathelicidins, hepcidins, peptides derived from histones and piscidins, can be found in fish [68].

These peptides work as general protective mechanisms in fish skin, do not induce toxicity in the host and, thanks to a wide spectrum of antimicrobial activity, they are able to target various pathogens. Moreover, since they have a low specificity in most cases, the risk of developing resistance towards them is also very low. These peptides are non-specific compounds belonging to the innate immune system with a fundamental role in defense mechanisms. Antimicrobial peptides mainly consist of 12–45-amino acid chains with positive charge [69] and are mainly based on amphipathic secondary structures, like α-helices and β-sheets. When they are attached onto the outermost cover of the negatively charged membrane of bacteria, with the help of electrostatic and hydrophobic connections, they can destroy the bacteria through biological and physiochemical methods. To date, several studies have been carried out on the action of these peptides against both Gram-positive and Gram-negative bacteria, highlighting that the host defense occurs mainly thanks to the formation of pores in the bacterial membranes and their subsequent destruction. The activity of antimicrobial peptides can be understood through the use of different models, including the toroidal pore model, barrel stave model, Shai Matsuzaki Huang model and carpet model. Many other hypothetical and tentative models have been used to understand the action of antimicrobial peptides in order to show how they can break cell membranes, involving dynamic simulations, molecular mean-field theory and coarse-grained simulations [70].

Several fish families have body parts rich in peptides with antimicrobial activity, such as pleurocidins, including red sea bream, American plaice, *Pleuronectes americanus*, Atlantic halibut, *Hippoglossus hippoglossus* and *Hippoglossoides platessoides* [4,71]. The recovery of these peptides from fish side streams differ among fish species. For example, in hagfish, these peptides are separated from the intestine, while in red sea bream, they are produced from gills [15], and in striped bass, they are derived from gills and skin [16]. For example, the antimicrobial activity of peptides isolated from gills of *Chrysophrys* (red sea bream), in the form of three peptides, is similar to some extent in sequence but not identical to some originating from different RNA. These are amidated peptides from the C-terminus, known as chrysophsin-1, with 25 amino acids, chrysophsin-2 with 25 amino acids and chrysophsin-3 with 20 amino acids, and containing abundant positive charge [15].

### 4.4. Anticoagulant Effect

Anticoagulant peptides are mainly obtained through the enzymatic breakdown of proteins from fish muscle. Previous experiments carried out on a laboratory scale evaluating the antiplatelet and anticoagulant characteristics of these peptides showed they acted following the basic method to prevent clotting [58]. For example, an anticoagulant peptide with a molecular weight of 12.2 kDa was isolated from yellowfin sole fish. This peptide forms an inactive compound and stop the function of the XII clotting element. For antiplatelet activity, these peptides capture platelet accumulation by boosting glycoprotein integrin of platelet membranes. Another anticoagulant peptide with a molecular weight of 3.34 kDa was isolated from *Urechis unicinctus* (order *Urechide*). Its action on blood factor IXa (FIXa) was observed, resulting in a noticeable prolongation of blood activated partial thromboplastin time (APTT).

### 4.5. Antiproliferative Effect

In the modern world, cancer is a main cause of death. It is caused by DNA mutations which disturb normal cell propagation methods and eventually lead to death. When cell monitoring is destroyed, undesired cell bodies are produced called a tumor. This tumor can also reach distant parts of the body [84]. It was reported that amino acids, proteins and peptides demonstrated antiproliferative or antitumor functions but the research carried out on antiproliferative peptides is scarce. Many antitumor peptides were discovered and isolated from seafood, like half-fin anchovy [85] and tuna dark muscle [86]. For example, the production of antiproliferative peptides through the breakdown hydrolysis of protein from tuna dark muscle by applying protease XXIII and papain enzymes was evaluated [86]. Both peptides have a molecular weight from 1.4 to 390 kDa. The peptide produced from protease XXIII enzyme had an amino acid sequence with a molecular weight of 1.12 kDa, including Glu–Gly–Gly–Val–Tyr–Met–Val–Thr amino acids. Another peptide produced from the papain enzyme had a 1.21 kDa molecular weight with a sequence of amino acids including Leu–Pro–His–Val–Leu–Thr–Pro–Glu–Ala–Gly–Ala–Thr. Their antiproliferative effect on the MCF-7 cell line was affected by the amount of peptide used, which required 8.8 mM of peptide isolated through the protease XXIII enzyme and 8.1 mM for peptide isolated through the papain enzyme [86]. Some scientists discovered peptides from salmon, cod, plaice and blue whiting that can inhibit the proliferation of two cell lines related to human breast cancer (MDA-MB-231 and MCF-7/6 cell lines) [87]. These peptides consisted of a complicated combination of amino acids with a molecular weight up to 7 kDa, including small amounts of sodium chloride and lipids. The antioxidant properties attributed to peptides are also related to their antiproliferative effect, since free radicals produced during the oxidation process become harmful for proteins and DNA, which leads towards tumor growth [84].

### 4.6. Calcium-Absorbing and Bone Mineralization Ability

Bioactive peptides have shown health effects on bones through the use of different animal models of osteoporosis and fractures [88]. It was documented that the absorption of calcium can be increased by using seafood peptides, because they contained hormone-like properties and some developmental regulators [89]. Marine peptides can be also used for the treatment of bone weakness and also for Paget’s disease by attaching on the surface of cell receptors on osteoclasts, which reduces the chance of osteoclast formation by promoting calcium metabolism [90,91].

## 5. Applications of Seafood-Derived Peptides and Industrial Relevance

Since many functional and bioactive properties have been widely reported for peptides recovered from fish side streams, potential effects on both human health and food quality are expected from these bioactive compounds. Therefore, pharmaceutical and food industries are behind the main target applications for these valuable compounds.

Marine-derived bioactive peptides have shown a high potential in the development of pharmaceutical products with an active role on human health. In this respect, capsules containing an oligopeptide obtained from albacore (*Thunnus alalunga*), which is further converted into its active form by digestive enzymes, have been produced as a tool for blood pressure monitoring [92]. Moreover, a recent combination of pharmaceuticals with cosmetics and seafood-derived bioactive compounds has become the hallmark of the cosmetic industry [93]. In this regard, the antioxidant properties associated with marine peptides are interesting for the development of antiaging and photo-protective cosmeceuticals. Many other applications have been recently reviewed elsewhere [93], highlighting the scientific evidence towards the enormous potential of fish peptides for the formulation of sustainable and effective cosmeceuticals.

Regarding the potential applications of marine-derived peptides in the food industry, their role as food stabilizers can be highlighted [94]. These peptides can keep moisture in food and have emulsion, foaming and structure stability properties [30]. As mentioned before, the foaming and emulsion properties of peptides can be monitored by optimizing their molecular weight or sequence length [33,95].

On the other hand, seafood-derived peptides could be used as substitutes for artificial or chemical preservatives [45] since they have been reported to stop food spoilage and extend shelf life [46,47]. In this sense, many studies showed that marine-derived peptides can act as antioxidants and cryoprotectants [52,53,96,97,98,99,100]. Fish-derived peptides have been used to prevent lipid oxidation in seafood products. In this regard, peptides obtained from pollock fish skin, through enzymatic hydrolysis, have been applied to pink salmon fillets; a decreased Thiobarbituric Acid Reactive Substances (TBARS) value in the treated samples was observed after storage at −35 °C for four months [101]. Peptides obtained from silver carp proved to reduce lipid oxidation in sierra fish after frozen storage. Moreover, silver carp peptides prevented a decrease in docosahexanoic and eicosapentaenoic acids and preserved the endogenous content of α-tocopherol [102]. Therefore, fish peptides are good candidates to be used as preservatives in fish products.

The preservation of gel formation in fish products is another potential application for peptides derived from seafood by-products. Many food products use surimi as a basic constituent [52]. Essential muscle proteins give a gel-forming quality to surimi. During the freezing process of surimi, two sugars, sucrose and sorbitol, are used to reduce protein deterioration produced by crystallization [53] but, as a result, these sugars give undesirable sweetness to the food [54]. To avoid this problem, peptides can be used to replace the sugars. Many studies proved that protein gel formation can be maintained by using peptides of seafood by-products. In this regard, shrimp head-derived protein hydrolysates have been suggested as good natural cryoprotectants to stabilize the denaturation of lizardfish protein and to improve the gel-forming capacity of lizardfish surimi under frozen storage [103].

## 6. Conclusions

Many biologically active molecules, such as fish protein hydrolysates and bioactive peptides, can be isolated from seafood side streams, which include fish muscle protein, fish skin collagen and gelatin, fish bone, fish internal organs and shellfish and crustacean shells, which are a rich source of protein. Different methods have been used for the extraction of bioactive peptides from fish proteins, although enzymatic hydrolysis with proteolytic enzymes has been applied the most. Both functional (solubility, emulsifying and foaming properties, water-holding or fat-binding capacity) and bioactive properties (antioxidant, antihypertensive, antiproliferative or antimicrobial) have been reported for marine-derived peptides, which highlight their current interest for the food and pharmaceutical industries. Although several studies have been conducted with regard to the extraction and purification, identification and characterization, structure and function of bioactive peptides, further studies are needed to determine the mechanisms that may mediate their biological activities and physiological effects. Moreover, it is crucial that future research evaluates their in vivo activity and efficacy in the human body.

## Figures and Tables

**Table 1:** Marine food by-products and their biological activities. Adapted from [72].

Marine Side Streams	Product	Enzyme Used	Activity	Reference
Smooth hound viscera (stomach and intestine)	Protein hydrolysate	Purafect, Esperase and Neutrase	Antioxidant, antihypertensive and antibacterial	[73]
Rainbow trout viscera (throat, stomach and intestines)	Protein hydrolysate	Pepsin	Antibacterial	[74]
Yellowfin sole frame	Bioactive peptide	Alcalase, a-chymotrypsin, mackerel intestine crude enzyme (MICE), Neutrase, papain, pepsin, pronase E, trypsin	Antioxidant	[75]
Alaska pollack frame	Bioactive peptide	MICE	Antioxidant	[76]
Jumbo squid skin	Bioactive peptide	Trypsin, a-chymotrypsin, pepsin	Antioxidant	[77]
Hoki frame	Bioactive peptide	Pepsin, trypsin, papain, a-chymotrypsin, alcalase, Neutrase	Antioxidant	[17]
Sardine heads and viscera	Bioactive peptide	Alcalase 2.4 L serine-protease from *B. licheniformis*, crude enzyme preparation from *A. clavatus*, alkaline proteases from *B. licheniformis*, crude enzyme extract from viscera of sardine (*Sardina pilchardus*)	Antioxidant	[78]
Tuna frame	Bioactive peptide	Alcalase, Neutrase, pepsin, papain, a-chymotrypsin, trypsin	Antihypertensive	[79]
Chum salmon skin	Bioactive peptide	Complex protease (7% trypsin, 65% papain, 28% alkaline proteinase)	Antioxidant	[80]
Salmon pectoral fin	Bioactive peptide	Pepsin	Anti-inflammatory	[81]
Black pomfret viscera	Bioactive peptide	Pepsin, trypsin, α-chymotrpsin	Antioxidant	[82]
Bluefin tuna head	Protein hydrolysate	*B. mojavensis* A21 proteases, alcalase from *B. licheniformis*	Antioxidant	[50]
Pacific cod skin	Bioactive peptide	Pepsin, trypsin, a-chymotrypsin	Antihypertensive and antioxidant	[83]
Sea rotifer	Bioactive peptide	Alcalase, a-chymotrypsin, Neutrase, papain, pepsin and trypsin	Antioxidant	[51]
Cod backbones	Protein hydrolysate	Proteolytic enzyme (Protamex)	Antioxidant	[52]
Squid by-products	Bioactive peptide	Protease type XIV	Antioxidant and antimutagenic	[55]
Squid by-products	Bioactive peptide	Endogenous proteases	Antihypertensive	[56]
Tuna bone	Bioactive peptide	Alcalase, a-chymotrypsin, Neutrase, papain, pepsin and trypsin	Antioxidant	[27]
Salmon trimmings	Bioactive peptide	Alcalase 2.4 L, Flavourzyme, corolase PP or promod 144 MG	Antioxidant, antihypertensive	[44]
Seabass skin	Protein hydrolysate	Alcalase	Antioxidant, antiproliferative and immunomodulatory	[45]
Unicorn leatherjacket skin	Protein hydrolysate	Glycyl endopeptidase	Antiproliferative, antiproliferative and immunomodulatory	[46]
Salmon skin	Bioactive peptide	Alcalase and papain	Antihypertensive	[47]
